# Abberant inverted U-shaped brain pattern and trait-related retinal impairment in schizophrenia patients with combined auditory and visual hallucinations: a pilot study

**DOI:** 10.1007/s11682-020-00281-y

**Published:** 2020-04-17

**Authors:** Chuanjun Zhuo, Bo Xiao, Ce Chen, Deguo Jiang, Gongying Li, Xiaoyan Ma, Ranli Li, Lina Wang, Yong Xu, Chunhua Zhou, Xiaodong Lin

**Affiliations:** 1 Department of Psychiatry Pattern Recognition, Department of Genetics Laboratory of Schizophrenia, School of Mental Health, Jining Medical University, Jining, 272119 Shandong Province China; 2 Department of Psychiatry, Wenzhou Seventh People’s Hospital, Wenzhou, 325000 China; 3 Department of Psychiatric-Neuroimaging-Genetics and Co-morbidity Laboratory(PNGC_Lab), Tianjin Anding Hospital, Tianjin Mental Health Center, Tianjin Medical University Mental Heath Teaching Hospital, Tianjin, 300222 China; 4 Department of Psychiatry, First Hospital/First Clinical Medical College of Shanxi Medical University, Taiyuan, China; 5 MDT Center for Cognitive Impairment and Sleep Disorders, First Hospital of Shanxi Medical University, Taiyuan, 030001 China; 6 Department of Psychiatry, Tianjin Medical University, Tianjin, 300074 China; 7 Department of Medical Big Data Centre, Shanxi Medical University, Taiyuan, China; 8 Department of OCT, Tianjin Eye Hospital, Tianjin, 300274 China; 9 Department of Pharmacoloy, The First Hospital of Hebei Medical Universtiy, Shijiazhuang, 05000 Hebei Province China

**Keywords:** Schizophrenia, Auditory hallucinations, Vision hallucinations, GMV, gFCD

## Abstract

Schizophrenic patients often experience auditory hallucinations (AHs) and visual hallucinations (VHs). However, brain and retinal alterations associated with combined AHs and VHs in schizophrenic patients are unknown. This study aimed o investigate brain and retinal alterations in first episode un-treated schizophrenic patients with combined AHs and VHs (FUSCHAV). FUSCHAV patients (*n* = 120), divided into four groups according to severity of AH and VH symptoms, were compared to healthy controls (*n* = 30). Gray matter volume (GMV) and global functional connectivity density (gFCD) were recorded to reflect brain structure and functional alterations. Total retinal thickness was acquired by optical coherence tomography to assess retinal impairment. The majority of FUSCHAV patients (85.8%) demonstrated both GMV reduction and gFCD increases along with retinal thinning compared to healthy controls. The severity of GMV reduction and gFCD increase differed between patient groups, ranked from highest to lowest severity as follows: severe AHs combined with severe VHs (FUSCHSASV, 20 patients), moderate AHs combined with severe VHs (FUSCHMASV, 23 patients), severe AHs combined with moderate VHs (FUSCHSAMV, 28 patients), and moderate AHs combined with moderate VHs (FUSCHMAMV, 26). Retinal impairment was similar among the four FUSCHAV groups. GMV reduction and gFCD increases in the frontal-parietal lobule show an inverted U-shaped pattern among FUSCHAV patients according to AH and VH severity, while retinal impairment remains stable among FUSCHAV groups. These findings indicate a reciprocal deterioration in auditory and visual disturbances among FUSCHAV patients.

## Introduction

Auditory perceptual disturbances in patients with schizophrenia are generally experienced as auditory hallucinations (AHs) (Pinheiro et al. 2019). The prevalence of AHs is greater than 40% in patients with schizophrenia (Upthegrove et al. 2016), and prevalence in first episode, un-medicated schizophrenic patients is even higher (Zhuo et al. 2019). Increasingly, studies have focused on the brain pathology associated with AHs and several hypotheses have been established (Baumeister et al. 2017; Blom 2015; Hugdahl and Sommer 2018; Northoff 2014). While these hypotheses have not been met with full acceptance, these prior studies have provided many important findings on the brain mechanisms associated with AHs (Huang et al. 2019; Stephane 2019; Zmigrod et al. 2016).

Visual perceptual disturbances in schizophrenia are experienced as visual hallucinations (VHs) and vision distortions (Green et al. 2012). The prevalence of VHs in schizophrenic patients is approximately 25–30% (Waters et al. 2014). Prior studies have reported that VHs are experienced in multiple stages of schizophrenia, and has also been reported in children, adolescents, and young adults at high-risk for schizophrenia (Grano et al. 2015; Hebert et al. 2010; Mittal et al. 2015). Furthermore, according to some reports, visual disturbances can be used as an index for symptoms, endophenotypes, biomarkers, and predictors for the study of the clinical, pathological and physiological features of schizophrenia (Garcia-Portilla et al. 2019; Guidotti and Grayson 2011).

In the last decade, imaging studies using both magnetic resonance imaging (MRI) and optical coherence tomography (OCT), have focused on brain and retinal aberrations in schizophrenia. Specifically, studies about the relationship between brain and retinal aberrations and the symptoms of visual disturbances have reported that patients with schizophrenia present with brain functional disturbances and retinal thickness impairment (Calderone et al. 2013; Lencer et al. 2005; Nagel et al. 2007; Onitsuka et al. 2007; Silverstein et al. 2009, 2010). These findings provide pivotal information for further study. The poor treatment outcome of schizophrenic patients with a combination of auditory and visual system disturbances support the above mentioned findings (Silverstein 2016). Hence, the current study investigated the pathological features of schizophrenic patients presenting with both AHs and VHs. Specifically, first-episode unmedicated patients were studied to avoid influnces of previous interventions.

Inspired by Silverstein (2016), who stated that without radical re-visioning of research ideas, it is difficult to achieve scientific advancement (Silverstein 2016), we conducted a pilot study to investigate pathological features of the first episode of un-medicated schizophrenic patients with combined auditory and visual disturbance. Unlike prior studies that separated patients into those experiencing visual disturbances and those experiencing auditory disturbances, the current study investigated whole-brain and retinal abberations in patients with a combination of both auditory and visual disturbances. Three hypotheses were tested. Our first hypothesis was that first episode untreated schizophrenic patients with AHs and VHs (FUSCHAV patients) would demonstrate brain structural/functional and retina alterations. Our second hypothesis was that brain and retinal alteration patterns would differ according to severity of AHs and VHs. Finally, our third hypothesis was that auditory and visual hallucinations may show reciprocal deterioration in FUSCHAV patients.

## Methods

Patients were recruited between January 2018 and July 2019 from five institutes (Tianjin Mental Heal Center, the School of Mental Health at Jining Medical University, Wenzhou Seventh People’s Hospital, Tianjin Kangtai Hospital, The First Hospital of Shanxi Medical University) to participate in the study. The ethics committee of all five institutes approved study methods. All MRIs and OCTs were conducted by one technician.

The inclusion criteria for FUSCHAV were as follows: 1) fully meets the DSM-IV diagnostic criteria for SCH; 2) first schizophrenia diagnosis in a mental health professional hospital; 3) no antipsychotic therapeutics for at least three weeks prior to the study; 4) simultaneously experiences AHs and VHs; 5) aged 18 to 30 years; 6) no substance abuse; 7) no other systemic disease, chronic disease, or head trauma; 8) no other diseases which can cause AHs and VHs; and 9) no other diseases which can cause retinal disease. Healthy controls were recruited from hospital staff and adult medical students. The healthy controls did not have any psychiatric disorders or first-degree relatives with psychotic disorders as assessed by two professional psychiatrists using the SCI-D NP version. The exclusion criteria for patients and healthy controls were as follows: 1) moderate to severe physical disease (e.g. respiratory, cardiovascular, endocrine, neurological, liver, or kidney disease); 2) currently receiving electroconvulsive therapy; 3) a history of loss of consciousness for more than five minutes for any cause; 4) left-handedness, as determined by the Annett Hand Preference Questionnaire; 5) ophthalmic disease; 6) high myopia; 7) any magnetic resonance imaging (MRI) contraindication, including claustrophobia; and 8) IQ < 80.

### MRI data acquisition

The 3.0-Tesla MR system (Discovery MR750, General Electric, Milwaukee, WI, USA) was used to collect MRI data. Functional magnetic resonance imaging (fMRI) was performed using a GE Healthcare Discovery MR750 3 T MRI system (General Electric, Milwaukee, WI, USA) with an eight-channel phased-array head coil. Participants lay in a supine position and were asked to restrict thoughts and head movements during imaging. The imaging parameters were as follows: 2000 ms repetition time (TR), 45 ms echo time (TE), 32 slices, 4 mm slice thickness, 0.5 mm gap, field of view (FOV), 64 × 64 acquisition matrix, and 90° flip angle. SENSitivity Encoding (SENSE), with a SENSE factor of 2 and parallel imaging were used for all scans. Images were obtained with a high-resolution, three-dimensional turbo-fast echo T1-weighted sequence with the following parameters: 8.2/3.2-ms TR/TE, 188 slices, 1 mm thickness, no gap, 256 × 256 FOV, 256 × 256 acquisition matrix, and 12° FA.

### OCT data acquisition

OCT data were acquired with the OCT 4000 system (Zeiss, Germany (premierop.com/zeiss-cirrus-hd-oct-4000-used)) which collects retinal scans of both eyes of participants. The hand-held probe was mounted and subjects were positioned on a chin-head rest and asked to focus on a fixation target. A 5-s volumetric 10 mm × 5 mm scan of the foveal center, marked by outer segment layer (OSL) thickening, was captured. Each scan consisted of 500 A-scan/B-scans, 50 B-scans and five frames/B-scan, with acceptable scans containing ≥ five consecutive B-scan frames of the foveal center with no movement artefacts.

### Psychotic, auditory and visual hallucination symptoms severity assessment

Total severity of schizophrenia was assessed by the Positive and Negative Symptoms Scale (PANSS) (Fleischhacker et al. 2019). The scale for the Assessment of Positive Symptoms (SAPS) (Kumari et al. 2017) was used to measure the severity of AHs and VHs. Cognitive ability was assessed with the MATRICS Consensus Cognitive Battery (MCCB) (Lees et al. 2015). The Global Assessment of Functioning (GAF) was used to assess global function (Gspandl et al. 2018).

Patients were categorized into four groups according to the severity of AHs and VHs as measured by the SAPS items of visual and auditory hallucination. Scores of AHs and VHs above 4 were defined as severe. Patients who scored between 2 and 3 on AHs and VHs were defined as middle to moderately severe AHs and VHs, respectivey. According to the above classification rule, we established four groups: severe AHs combined with severe VHs (FUSCHSASV, 20 patients); middle to moderate AHs combined with severe VHs (FUSCHMASV, 23 patients); severe AHs combined with middle to moderate VHs (FUSCHSAMV, 28 patients); and middle to moderate AHs combined with middle to moderate VHs (FUSCHMAMV, 26 patients).

### sMRI data processing

Analysis of differences in brain volume was performed by voxel-based morphometry (VBM), using SPM8 (Statistical Parametric Mapping; v5; Institute of Neurology, London, UK). Bias correction, spatial normalization, segmentation into gray and white matter, imaging of cerebrospinal fluid, and intensity modulation were performed on 3D-FSPGR images in native space using SPM8. The DARTEL (Diffeomorphic Anatomical Registration Through Exponential Lie Algebra) toolbox (as proposed by Ashburner,) was used in a high-dimensional normalization protocol. Intensity modulation was performed by multiplying voxel values of the segmented images by the measure of warped and unwarped structures derived from the nonlinear step of the spatial normalization. During this step, relative regional gray matter density was converted into absolute gray matter density, and expressed as the amount of gray matter per unit volume of brain tissue before spatial normalization. The resulting modulated gray and white matter images were smoothed with a 6 mm Gaussian kernel. A multiple pattern recognition analysis was used to regress out covariate influences on gray matter. Significance threshold was set at *p* < 0.05, FEW-corrected. Covariates were: age, gender, education level, symptoms severity, GAF scores, and MCCB scores.

### fMRI data pre-processing

Resting-state fMRI scans were processed using Statistical Parametric Mapping 8 (SPM8; http://www.fil.ion.ucl.ac.uk/spm). The first 10 scan volumes were discarded to allow stabilization of the scanner and to allow patients to acclimate to the testing situation. The remaining volumes were corrected for slice timing and motion artifacts. Allowable motion thresholds (translational and rotational motion <2 mm and 2°, respectively) were checked for all fMRI data. Six of the motion parameters and the average blood oxygen level-dependent signals of the ventricles and white matter were removed. Data with specific-volume framewise displacement values >0.5 were excluded from analysis. Bandpass frequencies ranging from 0.01 to 0.08 Hz were used to filter data. Individual structural images were co-registered to the mean functional image, and the transformed structural images were co-registered to the Montreal Neurological Institute (MNI) space using linear registration. The motion-corrected functional volumes were spatially normalized to the MNI using parameters estimated during linear co-registration. Finally, the functional images were re-sampled into 3 mm cubic voxels for further analysis.

### Global functional connectivity density (gFCD) calculation

The gFCD was calculated for each voxel using a customized Linux script (Zhuo et al. 2014). A Pearson’s linear correlation was used to explore functional connectivity between voxels, with a correlation coefficient threshold of *r* > 0.6. Only voxels within the cerebral grey-matter mask were used in calculations of gFCD, and the gFCD for any given voxel (×0) was calculated as the total number of functional connections [k(×0)] between ×0 and all other voxels using a growth algorithm. This procedure was repeated for all voxels. To increase normality of the distribution, each gFCD value was divided by the mean value of all included voxels. A 6 × 6 × 6 mm^3^ Gaussian kernel was used to spatially smooth the gFCD maps to minimize the impact of anatomical differences between participants (Zhuo et al. 2014).

### OCT data analysis

A blind process of manual segmentation of individual retinal layers was performed by allocating random numbers to B-scan images prior to analysis. An ImageJ macro (http://imagej.nih.gov/ij/) (Jerotic et al. 2019) was used for segmentation. Average individual and combined layer thickness measurements were extracted from three macular regions relative to the foveal center (0 μm): (1) foveal region = −750 to 750 μm, (2) nasal parafoveal region = −1500 to −750 μm and (3) temporal parafoveal region = 750 to 1500 μm.

### Statistical analyses

One-way analyses of variance (ANOVA) were used to analyse demographic and clinical characteristics of participants (McHugh 2011). The Mann-Whitney test was used to compare GMV and gFCD between groups (Matsouaka and Betensky 2015). Gender differences were explored using Chi square analysis between two groups (Thomas 1990), correcting for GMV and gFCD. A *p* value <0.05 was considered significant.

## Results

### Demographic and clinical characteristics of participants

MRI data from five patients and OCT data from two patients were excluded from analysis due to poor acquisition quality. Among the remaining 113 patients, 109 patients demonstrated brain gray matter reduction and 97 patients demonstrated brain and retina co-structural impairment. Data from these 97 patients were used for analysis. Two independent psychiatrists with no involvement in the current study tested the validity of patient subtype divisions using the Auditory Hallucination Rating Scale (Haddock et al. 1999) and the PANSS P3 VHs (Fleischhacker et al. 2019) item to distinguish AH and VH severity. The four resulting symptom combination groups and diagnostic assessments of the psychiatrists affirmed our artificial divisions. While the patient groups significantly differed compared to healthy controls on MCCB and GAF, there were significant differences in these measures among the four patient groups. Detailed information is listed in Table 1.

**Table 1:** Demographic and clinical characteristics of participants

Variable	HCs *n* = 30	FUSCHSASV *n* = 20	FUSCHMASV *n* = 23	FUSCHSAMV *n* = 28	FUSCHMAMV *n* = 26	*F*	*P*
Age, years Mean (SD)	25.4(0.5)	22.0(4.2)	26.4(3.0)	25.2(1.2)	27.9(3.9)	63.40	<0.001
Gender (Femal/Male)	15/15	9/11	11/12	14/14	11/15	24.23	<0.001
Education level, years Mean (SD)	16.1(2.5)	14.5(2.0)	15.0(3.5)	14.5(2.5)	15.5(1.5)	23.12	<0.001
Duration of illness, Months, Mean (SD)	N/A	2.4(1.8)	3.2(2.0)	4.3(1.5)	6.2(2.7)	47.21	<0.001
PANSS score Mean (SD)	N/A	78.9(1.5)	80.1(6.8)	79.5(5.9)	78.6(9. 9)	127.73	<0.001
SAPS-AHs	N/A	4.6(0.2)	2.2(0.4)	4.5(0.1)	1.9(0. 4)	27.73	<0.001
SAPS-VHs	N/A	4.2(0.3)	3.7(0.2)	2.0 (0.5)	2.1 (0.3)	21.56	<0.001
GAF	100.0(0.0)	78.0(13.5)	72.0(10.5)	76.5(6.7)	80.2(9.9)	45.13	<0.001
MCCB score Mean (SD)							
Speed Processing	48.0(4.5)	30.1(8.5)	36.5(7.0)	38.5(8.0)	40.3(8.0)	99.66	<0.001
Attention	47.5(11.5)	20.3(4.3)	22.0(2.7)	23.8(6.5)	34.0(2.0)	100.10	<0.001
Working Memory	50.0(11.2)	21.2(4.6)	24.0(5.2)	34.0(4.3)	30.2(8.2)	98.96	<0.001
Verbal Learning	49.50(5.0)	30.4(5.5)	33.2(9.4)	37.4(2.5)	32.2(8.4)	77.52	<0.001
Visual Learning	45.0 (9.0)	24.0(3.3)	29.2(4.8)	30.0(4.1)	32.2(2.3)	111.10	<0.001
Problem Reasoning	45.6(4.5)	30.0(7.3)	32.0(6.5)	35.5 (7.3)	37.0(10.2)	93.89	<0.001
Social Cognition	47.5(1.5)	30.5(10.0)	34.4(8.2)	33.4(8.5)	36.9(12.0)	112.99	<0.001

### GMV reduction

**Fig. 1:**
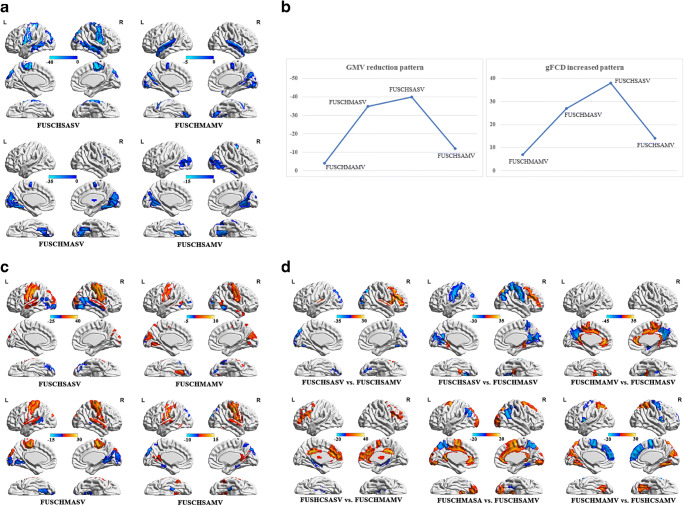
GMV and gFCD in patients. **a** Location of GMV reduction by patient group. **b** The inverted U-shape pattern of the GMV and gFCD alterations. X axis, types of disease combination; Y axis, peak value. **c** gFCD alterations in FUSCHAV patients. **d** Alterations in GMV volume (upper) and gFCD (lower) among patient groups (comparison between two groups)

Compared to the healthy control group, all four patient groups demonstrated GMV reduction. GMV reduction was mainly observed in the temporal, frontal, parietal, and occipital lobes with the greatest extent of GMV reduction located in the frontal-parietal lobe across all four groups (Fig. 1a). The four groups ranked by the extent of GMV reduction according to the peak value of reduction in the frontal-parietal lobe is as follows: FUSCHSASV, FUSCHMASV, FUSCHSAMV, and FUSCHMAMV. The extent of GMV reduction demonstrates an inverted U-shape pattern (Fig. 1b).

### gFCD alterations

Compared to the healthy control group, all four patients groups demonstrated gFCD increase. As with GMV alterations, the increase in gFCD was mainly observed in the temporal, frontal, parietal, and occipital lobes (Fig. 1c) with the greatest extent of gFCD increase in the frontal-parietal lobe across all four patient groups. The four groups ranked by the extent of gFCD increase in the frontal-parietal lobe according to peak value (greatest increase to lowest) is as follows: FUSCHSASV, FUSCHMASV, FUSCHSAMV, and FUSCHMAMV. As with GMV decrease extent, the extnet of gFCD increase demonstrates an inverted U-shape pattern (Fig. 1b).

### GMV differences among the four patient groups

Alterations in GMV volume were compared among patient groups (Fig. 1d, top). While the extent of GMV volume alterations in some brain regions differed between patient groups, the largest GMV alterations remained in the frontal and parietal lobes.

### gFCD alterations among the four patient groups

gFCD alterations were compared among all patient groups. While the extent of gFCD alterations in some brain regions differed between patient groups, the largest gFCD alteration were also mainly located in the frontal, parietal, and occipital cortices (Fig. 1d, bottom).

### Retinal alterations

**Fig. 2:**
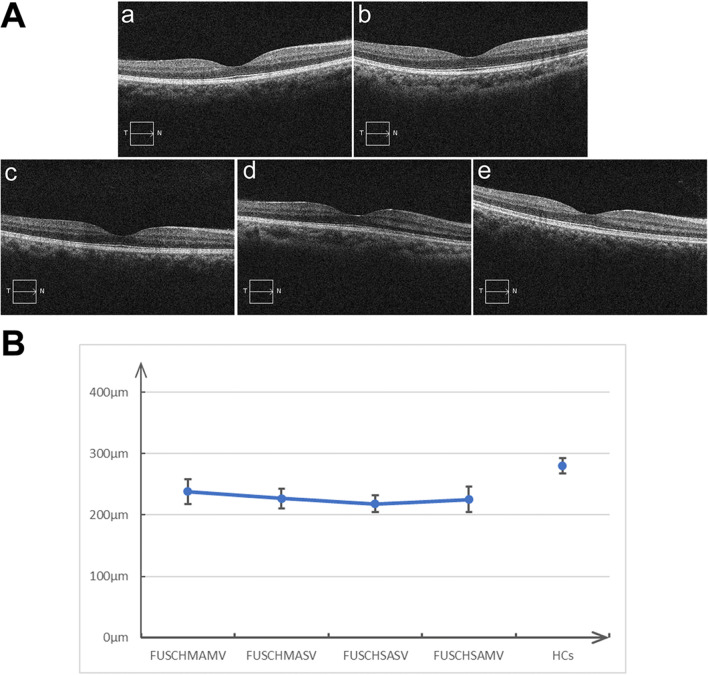
Total retinal thickness and the pattern of retinal thinning in patients and healthy controls. **A** The maximum impairment of total retinal thickness in FUSCHSASV (a), FUSCHMASV (b), FUSCHSAMV (c), and FUSCHMAMV (d) and the minimum total retinal thickness of healthy controls (e). **B** The pattern of retinal thinning in patients and healthy controls

All four patient groups showed significant retinal nerve fiber layer (RNFL) thinning compared to the heathly control group (Fig. 2 and Table 2). While overall, the RNFL did not significanly differ among patient groups, there was a regional complex pattern of RNFL alterations among groups (Table 3).

**Table 2:** OCT total retinal thickness alterations among different groups

Variable		Temporal parafoveal region	Foveal region	Nasal para-foveal region
FUSCHSASV		276.0 μm ± 15.0 μm	250.9 μm ± 20.9 μm	310.3 μm ± 10.7 μm
FUSCHMASV		280.3 μm ± 28.3 μm	253.5 μm ± 15.0 μm	312.2 μm ± 14.5 μm
FUSCHSAMV		274.0 μm ± 19.5 μm	257.1 μm ± 16.5 μm	317.2 μm ± 25.4 μm
FUSCHMAMV		275.5 μm ±10.0 μm	254.5 μm ± 12.0 μm	310.5 μm ± 10.7 μm
Healthy controls (HCs)		315.5 μm ±15.0 μm	268.3 μm ± 25.0 μm	330.5 μm ± 11.5 μm
Four FUSCHAV groups vs. HCs	F	17.56	21.03	30.03
	*P*	<0.001	<0.001	<0.001
FUSCHSASV vs. FUSCHMASV	t-test	0.757	0.720	0.580
	*P*	0.456	0.780	0.611
FUSCHSASV vs. FUSCHSAMV	t-test	0.605	0.742	0.647
	*P*	0.576	0.631	0.599
FUSCHSASV vs. FUSCHMAMV	t-test	0.553	0.653	0.580
	*P*	0.428	0.524	0.413
FUSCHMASV vs. FUSCHMAMV	t-test	0.620	0.753	0.673
	*P*	0.413	0.786	0.527
FUSCHMASV vs. FUSCHSAMV	t-test	0.703	0.603	0.499
	*P*	0.670	0.570	0.553
FUSCHSAMV vs. FUSCHMAMV	t-test	0.996	0.569	0.666
	*P*	0.878	0.578	0.591

**Table 3:** RNFL alterations among FUSCHAV groups by region

Variable	Right eye, μm			Left eye, μm		
	Nasal	Temporal	Global	Nasal	Temporal	Global
FUSCHSASV	62.8 ± 12.0	78.5 ± 11.5	79.8 ± 6.3	63.2 ± 8.2	76.5 ± 14.2	76.2 ± 10.2
FUSCHMASV	64.8 ± 8.8	81.0 ± 12.3	80.2 ± 16.3	60.5 ± 11.7	63.8 ± 18.2	74.5 ± 11.1
FUSCHSAMV	65.0 ± 13.45	61.2 ± 7.9	78.5 ± 13.7	62.0 ± 14.9	72.5 ± 10.3	73.3 ± 9.3
FUSCHMAMV	63.5 ± 19.0	79.4 ± 13.8	79.3 ± 10.5	71.9 ± 8.5	77.7 ± 18.3	78.3 ± 10.5
FUSCHAV inter-group comparisons	F = −1.105	F = 8.985	F = −1.003	F = 5.553	F = 4.985	F = −0.523
	*P* = 0.589	*P* < 0.001	*P* = 0.680	*P* < 0.001	*P* < 0.001	*P* = 0.560
HCs	82.6 ± 12.5	88.8 ± 15.5	105.5 ± 4.7	84.5 ± 11.0	90.8 ± 8.9	100.5 ± 14.0
All FUSCHAV vs. HCs	F = 8.963	F = 10.012	F = 11.233	F = 10.363	F = 13.117	F = 15.200
	*P* < 0.001	*P* < 0.001	*P* < 0.001	*P* < .001	*P* < 0.001	*P* < 0.001

## Discussion

The present study investigated brain and retinal alterations in first episode schizophrenic patients with auditory and visual hallucinations. All four groups of FUSCHAV patients demonstrated GMV reduction, primarily located in the temporal, occipital, frontal, and parietal lobes. These findings indicate that the primary auditory and visual cortex are impaired in FUSCHAV patients (Bernardin et al. 2019; Csaszar et al. 2019; Moseley et al. 2018). The first episode of schizophrenic patients with congenital deficiency or serious brain damage is generally severe auditory and visual hallucinations. More notably, the GMV reduction in the frontal and parietal lobes suggests that cortex involved in higher integration is affected. The patients in the current study were experiencing their first episode of schizophrenia and were unmedicated and therefore, these findings suggest that GMV reduction was present prior to the psychotic episode (Bordier et al. 2018; Zmigrod et al. 2016) and that there may be a brain-based pathology associated with the onset of AHs and VHs. Moreover, FUSCHAV patients demonstrated increased gFCD in the same brain regions showing GMV reduction suggesting that functional hyperactivity may compensation for structural impairment (Cao et al. 2018; Collier et al. 2014; Csaszar et al. 2019; Kremlacek et al. 2016; Xu et al. 2016). Interestingly, FUSCHAV groups differed in GMV and gFCD alterations in some brain regions. These alterations may be a distiguishing pattern for different patient groups. In addition to alterations in brain gray matter and connectivity, total retinal thickness was decreased in FUSCHAV patients compared to healthy controls, suggesting brain and eye co-impairmentt.

There were three major findings that we will address. First, GMV reduction and gFCD increases in the frontal-parietal lobule of FUSCHAV patients demonstrates an inverted U-shape pattern according to severity combinations of AHs and VHs. This inverted U-shape pattern suggests that visual disturbances are accompanied by relatively severe brain structural impairment and relatively strong functional compensation. However, GMV alterations in FUSCHSASV are more severe than in FUSCHSAMV indicating that the severity of VHs may enhance structutral brain impairments and functinonal compensation. The second major finding suggests that auditory and visual disturbances may be reciprocal and reflected as inverted U-shaped patterns of GMV and gFCD. These findings provide new clues for the further study of the mechanisms of the interatction between AHs and VHs in schizophrenia. Third, although brain alterations in FUSCHAV patients demonstrated an inverted U-shape according to severity of AHs and VHs, the retinal impairment in these patients did not differ. This finding suggests that while combinations of different severities of AHs and VHs are related to brain alterations, retinal thickness may be more of a trait marker and not related to symptom severity. This idea is consistent with prior research in first episode un-medicated patients (Bernardin et al. 2019; Moseley et al. 2018; Thomas 1990). However, contrary to this theory, other studies have reported that retinal thickness and brain GMV reduction are affected by treatment with antipsychotics (Bordier et al. 2018; Cao et al. 2018; Collier et al. 2014; Csaszar et al. 2019; Kremlacek et al. 2016; Xu et al. 2016; Zmigrod et al. 2016). Further cohort studies are needed to fully characterize the dynamical trajectory of brain and retinal alterations, to further understand the reciprocal action between the severity of AHs and VHs in patients with schizophrenia.

### Limitations

There are several limitations to the current study that must be considered when interpreting the results. First, the method used to group patients in the current study is novel and therefore further research needs to confirm the validity of this method. We have started to investigate this method by observing the evolution of symptoms in 51 patients for 6-month after starting anti-psychotic medication. We found varying degrees of aleviation from AVs and VHs, with VHs showing greater treatment resistance than AHs. These observations lend support for the group devision method used in the current study. Second, the small sample size of each group limits the significance of the findings. Third, total retinal thickness was used to assess eye impairment. The retina was not divided into layers to more precisely clarify retinal impairment and as such, our results may not provide the full picture of retinal impairment in FUSCHAV patients. We lacked access to software that can reliably divide the retina into fine slices for a more in-depth analysis. We are currently applying to the Zeiss OCT Institute for access to software that will enable us to further divide the layers of the retina to increase resolution in a future study of FUSCHAV patients.

Fourth, we calculated correlational coefficients to investigate potential relationships between GMV/gFCD, retinal thickness, and clinical symptoms (as measured by severity of AHs/VHs). In agreement with prior research (Lui et al. 2009; Milev et al. 2003), there were no significant correlations found between GMV/gFCD and clinical symptoms. In addition, we found no significant correlations between retal thickness and clinical symptoms. Prior research investigating this topic has yielded inconsistent results. The lack of significant correlations between clinical symptoms and GMV/gFCD or retinal thickness may indicate that these alterations are related to trait aspects of symptoms rather than to symptom severity (Lui et al. 2009; Milev et al. 2003). Future studies should include a measure of clinical symptom traits. Fifth, an important limitation of the current study is the lack of a group of schizophrenic patients without AHs and VHs for comparison. Such a group would enable further characterization of pathological features in our study population. We are working on enrolling such a comparison group for a future study.

## Conclusion

Despite the above described limitation, the current study reports on three important findings: 1) GMV reduction and gFCD increases in the frontal-parietal lobule of FUSCHAV patients demonstrate an inverted U-shape pattern according to differences in AH and VH severity, indicating functional compensation correlated with volume reduction, 2) There appears to be reciprocal deterioration in auditory and visual disturbances among FUSCHAV patients as observed through both brain and retinal abberations, 3) Although brain alterations in FUSCHAV patients differ with degree of AH and VH severity, retinal impairment remains constant among FUSCHAV groups.
